# Structure–function correlates of vision loss in neuromyelitis optica spectrum disorders

**DOI:** 10.1038/s41598-022-19848-4

**Published:** 2022-10-20

**Authors:** Norman K. Gigengack, Frederike C. Oertel, Seyedamirhosein Motamedi, Charlotte Bereuter, Ankelien Duchow, Rebekka Rust, Judith Bellmann-Strobl, Klemens Ruprecht, Tanja Schmitz-Hübsch, Friedemann Paul, Alexander U. Brandt, Hanna G. Zimmermann

**Affiliations:** 1 Experimental and Clinical Research Center, A Cooperation Between the Max Delbrück Center for Molecule Medicine and Charité - Universitätsmedizin Berlin, Corporate Member of Freie Universität Berlin and Humboldt-Universität zu Berlin, Lindenberger Weg 80, 13125 Berlin, Germany; 2 NeuroCure Clinical Research Center, Charité - Universitätsmedizin Berlin, Corporate Member of Freie Universität Berlin and Humboldt-Universität zu Berlin, Berlin, Germany; 3 Department of Neurology, University of California San Francisco, San Francisco, CA USA; 4 Department of Neurology, Charité - Universitätsmedizin Berlin, Corporate Member of Freie Universität Berlin and Humboldt-Universität zu Berlin, Berlin, Germany; 5 Department of Neurology, University of California, Irvine, CA USA; 6 Einstein Center Digital Future, Berlin, Germany

**Keywords:** Multiple sclerosis, Eye manifestations, Multiple sclerosis

## Abstract

Optic neuritis (ON) in neuromyelitis optica spectrum disorders (NMOSD) regularly leads to more profound vision loss compared to multiple sclerosis (MS) and myelin-oligodendrocyte-glycoprotein-antibody associated disease (MOGAD). Here we investigate ON-related vision loss in NMOSD compared to MS and MOGAD in order to identify neuroaxonal and retinal contributors to visual dysfunction. In this retrospective study we included patients with aquaporin-4-antibody seropositive NMOSD (n = 28), MOGAD (n = 14), MS (n = 29) and controls (n = 14). We assessed optic nerve damage and fovea morphometry by optical coherence tomography. Visual function was assessed as high (HCVA) and low contrast visual acuity (LCVA), and visual fields' mean deviation (MD). In all diseases, lower visual function was associated with peripapillary retinal nerve fiber layer (pRNFL) and ganglion cell and inner plexiform layer (GCIP) thinning following a broken stick model, with pRNFL and GCIP cutoff point at ca. 60 µm. HCVA loss per µm pRNFL and GCIP thinning was stronger in NMOSD compared with MOGAD. Foveal inner rim volume contributed to MD and LCVA in NMOSD eyes, only. Together these data supports that visual dysfunction in NMOSD is associated with neuroaxonal damage beyond the effect seen in MS and MOGAD. A primary retinopathy, respectively Müller cell pathology, may contribute to this effect.

## Introduction

Optic neuritis (ON) is a frequent manifestation of neuroinflammatory diseases such as neuromyelitis optica spectrum disorders (NMOSD), myelin oligodendrocyte glycoprotein (MOG)-IgG associated diseases (MOGAD) and multiple sclerosis (MS)^1–3^. ON causes neuroaxonal damage in the optic nerves and retina, which is associated with visual function loss^4^. ON in NMOSD regularly leads to more severe visual dysfunction than ON in MOGAD and MS^5^. This worse outcome can only in part be explained by more severe neuroaxonal damage assessed by macular ganglion cell and inner plexiform layer (GCIP) thinning^5^.

Pathogenic aquaporin-4 antibodies (AQP4-IgG) are specific for NMOSD and can be detected in the majority of patients^6–8^. AQP4 is an astrocytic water channel and expressed in retinal Müller cells^9^ with the highest concentration around the fovea centralis^10^. Foveal thinning^11,12^ and foveal shape changes^13^ have been described in AQP4-IgG seropositive NMOSD. Additionally, Müller cell dysfunction has been demonstrated through a decrease of specific b-wave amplitude in electroretinogram (ERG) tests of AQP4-IgG seropositive NMOSD patients^10^.

However, it is unclear if AQP4-IgG-driven loss and/ or dysfunction of Müller cells in the fovea may contribute to visual impairment in AQP4-IgG seropositive NMOSD associated ON. The aim of this study was to investigate the relationship between visual function, neuroaxonal damage, and foveal structure in AQP4-IgG seropositive NMOSD compared with MOGAD and MS.

## Methods

### Patients and controls

For this cross-sectional monocentric study, we retrospectively analyzed data from ongoing cohort studies of patients with autoimmune neuroinflammatory diseases. Inclusion criteria were AQP4-IgG or MOG-IgG seropositivity in at least one assay based on cell based assays^14,15^ (CBA) (AQP4-IgG: CBA, Euroimmun, Lübeck, Germany; MOG-IgG: by established cell-based assays using the laboratory’s cut-offs (MOG IFT, EUROIMMUN, Laboratory Stöcker, Germany; Molecular Neuroimmunology Group, University Heidelberg, Heidelberg, Germany)^16^, diagnosis of relapsing–remitting MS, or healthy controls. Exclusion criteria for all subjects were age below 18 or above 70, diagnosis of other relevant ocular diseases such as macular holes, amblyopia, glaucoma or hyperopic/myopic eyes of more than ± 5 dpt, and a timeframe of less than 3 months since the last ON episode. We included 28 AQP4-IgG seropositive NMOSD patients, 14 MOGAD patients, 29 MS patients, and 14 HCs. All AQP4-IgG seropositive and MS patients fulfilled the diagnostic criteria for NMOSD or MS, respectively, at the time of inclusion^7,17^. Of the 14 MOGAD patients, 5 fulfilled the clinical criteria for AQP4-IgG seronegative NMOSD^7^. In each case both eyes were included except for two eyes of the NMOSD and MOGAD group each, which were excluded for unrelated ocular disease. MS patients were not systematically tested for presence of AQP4- or MOG-IgG. All patients underwent scoring with the expanded disability status scale (EDSS)^18^.

### Ethics

NMOSD/MOGAD and MS/HC cohort studies were approved by the ethics committee of Charité – Universitätsmedizin Berlin (EA1/041/14 and EA1/163/12) and conducted according to the Declaration of Helsinki in its currently applicable version. All participants gave written informed consent.

### Optical coherence tomography

All patients and controls underwent optical coherence tomography (OCT) using Spectralis OCT (Heidelberg Engineering, Heidelberg, Germany) with automatic positioning system. Thickness of the peripapillary RNFL (pRNFL) was measured in 3.5 mm ring scans around the optic nerve head as automatically positioned by the device. Thickness of the combined ganglion cell and inner plexiform layer (GCIP) was acquired from the volume of a macular cylinder centered on the fovea within a 6 mm diameter. In the case of peripapillary ring scans, automated pRNFL segmentation was carried out by the device-internal software (Heidelberg Eye Explorer Version 6.3). Automated segmentation of macular volume scans was performed with the SAMIRIX pipeline^19^, based on OCTLayerSegmentation^20^, part of AURA Tools on NITRC (https://www.nitrc.org/projects/aura_tools/). Scan quality and automated segmentation was checked^21^ and corrected by two trained raters (NG, FCO). All raters were masked for the patients’ clinical and visual function characteristics. Furthermore, we used fovea morphometry previously described in detail to model and extract foveal shape parameters from the corrected macular volume scans^22^. We analyzed the inner rim volume, which is calculated in a 0.5 mm radius around the foveal center between inner limiting membrane (ILM) and Bruch’s Membrane (BM). Inner rim volume reflects volume of the outer retina including photoreceptors at the fovea and demonstrated strong differentiation between AQP4-IgG seropositive NMOSD and MS in a previous study^13^. OCT methods are reported in line with the APOSTEL recommendations^23^.

### Visual function

All patients and controls underwent monocular vision assessment for both eyes. Best corrected visual acuity was tested with retro-illuminated Early Treatment Diabetic Retinopathy Study (ETDRS) charts at a distance of 4 m for high contrast visual acuity (HCVA), and Sloan 2.5% contrast charts at a distance of 2 m for low contrast visual acuity (LCVA) (Precision Vision, La Salle, IL). For HCVA, if a patient was not able to identify any letters at 4 m, the distance was reduced stepwise to 0.5 m; for LCVA, the distance of 2 m was not changed. Visual acuity was measured as decimal acuity and then converted to the logarithm of the Minimum Angle of Resolution (logMAR). The worst possible HCVA was 1.9 logMAR. We excluded eyes that could not complete acuity testing due to vision poorer than that and did not assign a logMAR-value for finger counting, hand movement, and light perception. Visual field testing was performed under best correction in a 30-2 field with a Heidelberg Edge Perimeter (Heidelberg Engineering, Heidelberg, Germany) in a darkened room, using the SAP-III 30-2 ASTA protocol. We analyzed visual fields using the mean deviation (MD).

### Statistical methods

Statistical analysis was performed with R version 3.5.3^24^ using the packages ggplot2^25^, lme4^26^ and MuMIn^27^. Significance was established at *p* < 0.05. Group comparisons were done with Fisher´s Exact Test for categorical variables and with Mann-Whitney-U-tests in each group pair for continuous parameters.

We included eyes with and without history of ON into further analysis. The association between visual function and retinal layers was investigated with linear mixed models, accounting for within patient inter-eye-correlations as a random intercept. Non-standardized effect size is given as Beta ± standard error. To model the previously described steep drop in visual function below a certain threshold of neuroaxonal damage^28,29^ we used linear spline models^30^. In previous studies the threshold was identified to be at an approximate pRNFL thickness of 50–75 µm^29,31–33^. After visual evaluation of scatterplots of our data we determined that a cutoff at 60 µm for both pRNFL and GCIP thickness would be most appropriate to reflect the linear spline model. We then calculated the linear spline models with the knot at that cutoff for each patient group. In a sensitivity analysis, changing knot placement to 75 µm pRNFL thickness did not affect significance of results and only resulted in minor changes of effect size (data not shown). Furthermore, as there were only three eyes of MS-patients with a pRNFL thickness below 60 µm, we did not include the MS group in those comparisons. Differences in the relationship between visual function and retinal structure between all patient groups were examined using an interaction effect for diagnosis in the mixed models. Likewise, we investigated whether there was a relationship between visual function and foveal inner rim volume, as well as between inner rim volume and pRNFL thickness. As foveal thickness was shown to be lower in women^34^, and there was a mismatch of women and men between our AQP4-IgG seropositive and MOGAD cohorts, we additionally analyzed inner rim volume in eyes of only female participants. We did not perform post-hoc correction for multiple testing due to the exploratory nature of the study.

## Results

### Cohort characteristics

We analyzed 54 eyes of 28 AQP4-IgG seropositive NMOSD patients, 27 eyes of 14 MOGAD patients, 58 eyes of 29 MS-patients and 28 eyes of 14 HC (Table 1). Twenty-three eyes of the NMOSD group, 15 eyes of the MOGAD group and 24 eyes of the MS group had prior history of ON. NMOSD patients had a higher proportion of women compared with the MOGAD group and were older than the MS group. NMOSD patients also presented with a higher EDSS and visual functional system score than MS patients. Furthermore, NMOSD and MOGAD patient eyes with history of ON had a higher number of ON compared with MS eyes. There was no difference in time since first and last ON (per eye) for each group.

**Table 1:** Patient and eye characteristics.

		AQP4-IgG+	MOG-IgG+	MS	HC	AQP4-IgG+ versus MOG-IgG+	AQP4-IgG+ versus MS	AQP4-IgG+ versus HC
Subjects	n	28	14	29	14			
Sex Female	N (%)	26 (92.9%)	7 (50%)	21 (72.4%)	10 (71.4%)	**< 0.01**	0.08	0.16
Age [years]	Median [Range]	49.5 [20–69]	46.0 [21–59]	39.4 [25–64]	41.7 [24–68]	0.52	**0.01**	0.43
EDSS	Median [Range]	3.75 [0.00–6.50]	2.50 [1.00–6.00]	2.00 [0.00–4.50]	–	0.06	**< 0.01**	–
Visual FS	Median [Range]	1.00 [0.00–6.00]	0.00 [0.00–3.00]	0.00 [0.00–5.00]		0.07	**0.01**	
Eyes	N	54	26	58	28 (−)			
ON+ eyes	N_ON_	23	15	24				
ON History [Patients: NON/ON unilateral/ON bilateral]	N_NON_/N_uniON_/N_bilON_	12/9/7	4/5/5	10/14/5	–			
No. of ON (by ON-eye)	Median [Range]	2 [1–8]	2 [1–5]	1 [1, 2]	–	0.3	**0.02**	–
Years since first ON (by eye)	Median [Range]	6.9 [0.8–28.1]	6.3 [0.8–42.6]	6.5 [0.6–43.0]	–	0.48	0.77	–
Years since last ON (by eye)	Median [Range]	4.7 [0.3–28.1]	4.4 [0.3–39.6]	6.5 [0.3–43.0]	–	0.95	0.24	–

*AQP4-IgG*+ aquaporin-4 antibody seropositive patients, *MOG-IgG*+ myelin-oligodendrocyte-glycoprotein antibody seropositive patients, *MS* multiple sclerosis, *HC* healthy control, *SD* standard deviation, *EDSS* expanded disability status scale, *FS* functional system, *ON* optic neuritis.

*P*-values from Fischer´s Exact Test (sex ratio) or Mann–Whitney-U test (all other items); statistically significant results are printed in bold.

### Group comparisons of retinal structural and visual function parameters

**Figure 1:**
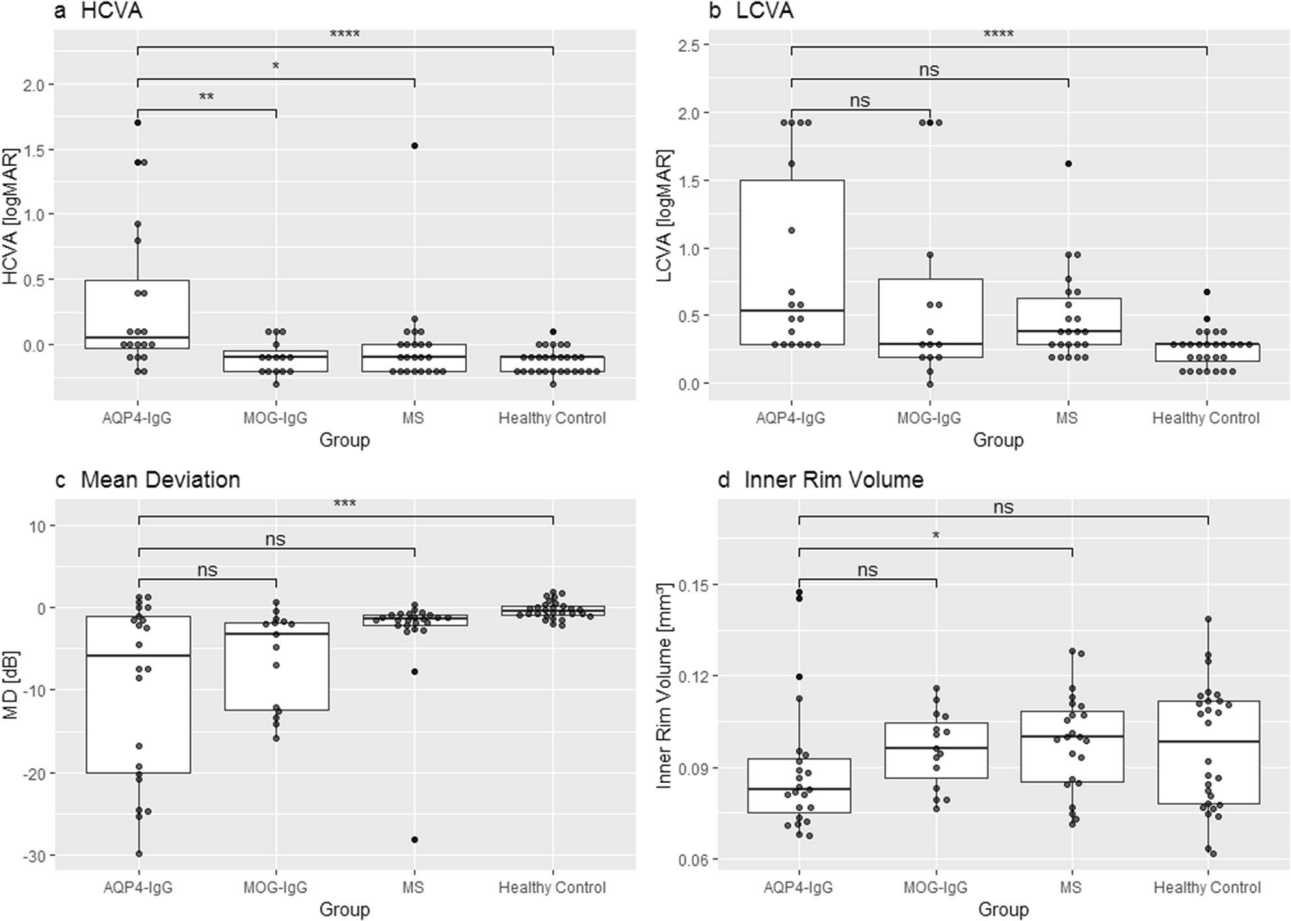
Group comparison of selected structural and functional parameters. (**a**) High contrast visual acuity (**b**) Low contrast visual acuity (**c**) mean deviation of visual fields and (**d**) inner rim volume of the fovea of eyes with history of ON AQP4-IgG: Aquaporin-4 IgG seropositive patients; MOG-IgG: Myelin-Oligodendrocyte-Glycoprotein seropositive patients; MS: Multiple Sclerosis; HCVA: High contrast visual acuity; LCVA: Low contrast visual acuity; logMAR: Logarithm of minimum angle of resolution; MD: mean deviation; p-values from Mann–Whitney-U Test (ns: *p* > 0.05; *: *p* < 0.05; **: *p* < 0.01; ***: *p* < 0.001; ****: *p* < 0.0001).

First, we compared visual function and OCT between NMOSD and other patient groups and HC (Table 2) and focusing on ON-eyes (Fig. 1). In summary, HCVA LCVA and MD in AQP4-IgG seropositive NMOSD were worse than in HC. While HCVA was worse in eyes of AQP4-IgG seropositive NMOSD with ON compared with MOGAD and MS, this was not significant for LCVA. MD was worse in ON-eyes of AQP4-IgG seropositive NMOSD compared with HC but not compared to MOGAD and MS patients. pRNFL and GCIP of AQP4-IgG seropositive NMOSD-ON-eyes were thinner compared with HC, but not to MOGAD. pRNFL of NMOSD-ON-eyes was significantly thinner than in MS-ON-eyes. Foveal inner rim volume was lower in ON-eyes of AQP4-IgG seropositive NMOSD patients when compared with MS and, in trend, the MOGAD group, but not to HC. NMOSD-eyes without history of ON also showed a significantly lower inner rim volume than MS and, in trend, MOGAD (Supplementary Fig. 1). When only counting females, inner rim volume was still not significantly different in NMOSD- and MOGAD-ON-eyes (p = 0.28).

**Table 2:** Structural and visual function parameters.

	AQP4-IgG+		MOG-IgG+		MS		HC	AQP4-IgG+-nON versus MOG-IgG + -nON	AQP4-IgG+-nON versus MS-nON	AQP4-IgG+-nON versus HC	AQP4-IgG+-ON versus MOG-IgG + -ON	AQP4-ON versus MS-ON	AQP4-IgG+-ON versus HC
	nON	ON	nON	ON	nON	ON	–						
	Mean (SD)							*p*-value					
HCVA [logMAR]	− 0.09 (0.11)	0.33 (0.59)	− 0.11 (0.15)	− 0.10 (0.13)	− 0.13 (0.11)	− 0.004 (0.36)	− 0.13 (0.09)	0.46	0.07	0.21	**0.004**	**0.013**	**< 0.001**
LCVA [logMAR]	0.29 (0.17)	0.85 (0.68)	0.25 (0.19)	0.65 (0.70)	0.31 (0.23)	0.48 (0.34)	0.26 (0.14)	0.59	0.69	0.36	0.15	0.07	**< 0.001**
MD [dB]	− 0.95 (2.36)	− 9.76 (10.6)	− 0.27 (0.85)	− 6.10 (5.83)	− 1.05 (1.41)	− 2.78 (5.63)	− 0.30 (1.05)	0.26	0.30	0.24	0.59	0.08	**< 0.001**
pRNFL [µm]	94.9 (11.5)	59.5 (23.4)	98.5 (13.0)	51.7 (13.5)	91.1 (11.9)	74.4 (15.6)	96.4 (10.1)	0.49	0.11	0.57	0.39	**0.01**	**< 0.001**
GCIP [µm]	65.6 (7.15)	51.7 (9.95)	69.2 (4.63)	50.6 (7.84)	66.6 (7.44)	56.0 (7.22)	68.7 (4.83)	0.11	0.72	**0.03**	0.61	0.13	**< 0.001**
Inner rim volume [mm^3^]	0.090 (0.015)	0.090 (0.022)	0.107 (0.024)	0.096 (0.012)	0.111 (0.018)	0.098 (0.016)	0.096 (0.021)	0.06	**< 0.001**	0.25	0.051	**0.02**	0.19

Test statistics from Mann–Whitney-U Test.

*AQP4-IgG*+aquaporin-4-IgG seropositive patients, *MOG-IgG*+myelin-oligodendrocyte-glycoprotein seropositive patients, *MS* multiple sclerosis, *nON* eyes without history of optic neuritis, *ON* eyes with history of optic neuritis, *SD* standard deviation, *HCVA* high contrast visual acuity, *LCVA* low contrast visual acuity, *logMAR* logarithm of minimum angle of resolution, *MD* mean deviation, *pRNFL* peripapillary retinal nerve fiber layer, *GCIP* combined ganglion cell and inner plexiform layer.

Significant values are in bold.

### Structure–function associations

**Figure 2:**
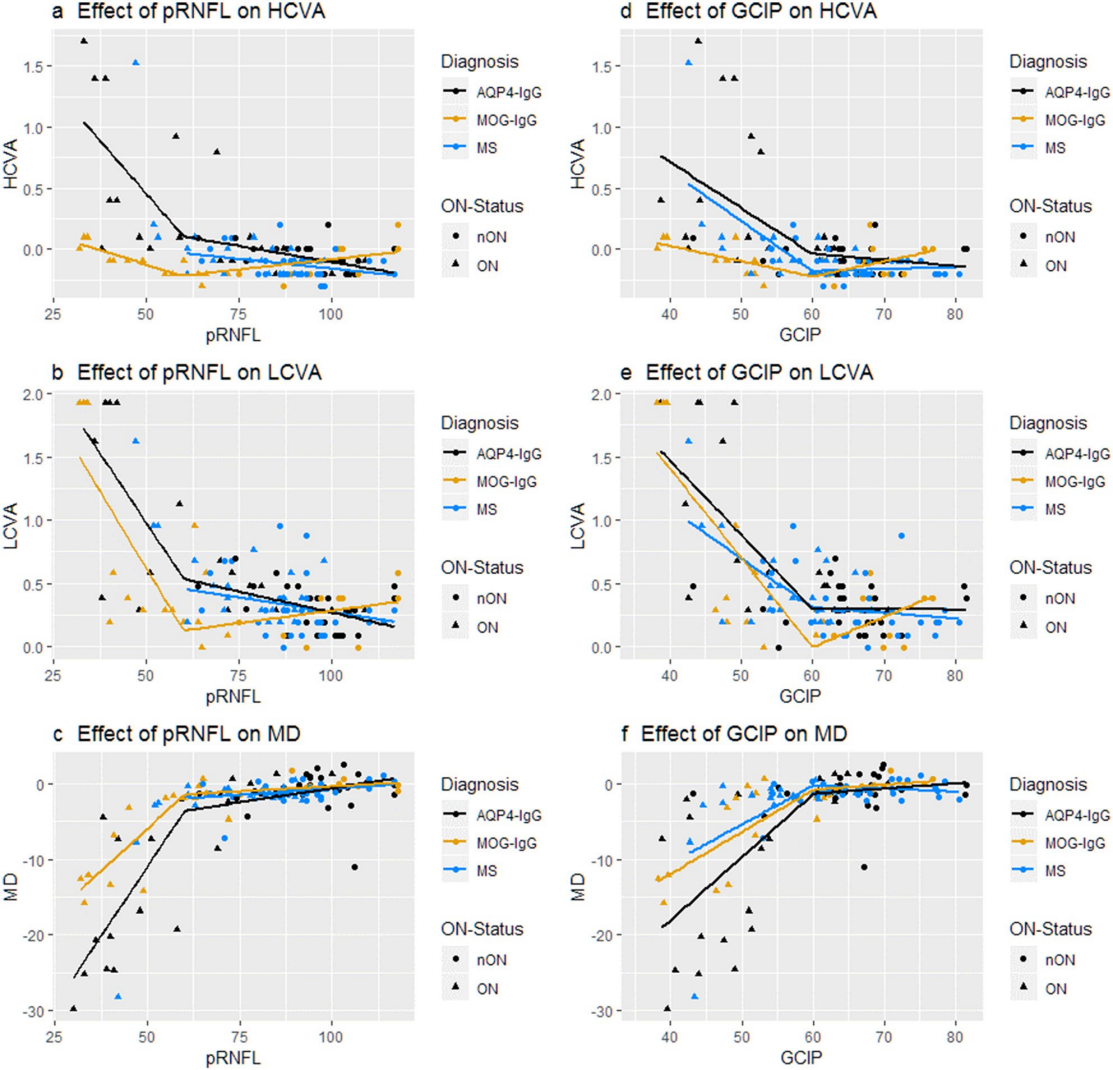
Association between visual function and retinal structures. (**a**–**c**): Effect of pRNFL thickness on (**a**) high contrast visual acuity, (**b**) low contrast visual acuity, (**c**) mean deviation of visual fields; (**d**–**f**): Effect of GCIP thickness on (**d**) high contrast visual acuity, (**e**) low contrast visual acuity (**f**) mean deviation of visual fields. Effects were modelled with a linear spline model; knot location was chosen to be 60 µm for both pRNFL and GCIP models following subjective visual assessment. AQP4-IgG: Aquaporin-4-IgG seropositive patients; MOG-IgG: Myelin-Oligodendrocyte-Glycoprotein-IgG seropositive patients; MS: Multiple Sclerosis patients; HCVA: High contrast visual acuity; LCVA: Low contrast visual acuity; MD: mean deviation; pRNFL: peripapillary retinal nerve fibre layer; GCIP: ganglion cell and inner plexiform layer.

We used mixed linear spline models to investigate the impact of changes in metrics of neuro-axonal damage on visual function in the different patient groups (Fig. 2). For a thickness of pRNFL or GCIP above 60 µm there was no significant effect on visual function in either group, except for a very small but formally significant association between pRNFL and LCVA in MS (Beta = − 0.004 ± 0.002; *p* = 0.05). Because of this, Table 3 only summarizes the results of the mixed spline models for a thickness of RNFL or GCIP below 60 µm (see Supplemental Table 1 for other results). Below 60 µm, thinner pRNFL and GCIP were associated with worse HCVA, LCVA and MD in all disease groups, respectively, with the exception of MS eyes with a pRNFL below 60 µm, which were not calculated due to low event number. In order to consider possible differences between patient groups regarding the rate to which structural changes affect visual outcome, we added the underlying diagnosis as an interaction effect in the mixed models. Further loss of pRNFL beyond a thickness of 60 µm caused a stronger impairment of HCVA and visual field MD in NMOSD-eyes compared with MOGAD-eyes, while there was no difference between the groups regarding LCVA.

**Table 3:** Structure–function associations for pRNFL and GCIP below 60 µm.

	AQP4-IgG+		MOG-IgG+		MS		Interaction AQP4-IgG+ versus MOG-IgG+		Interaction AQP4-IgG+ versus MS	
pRNFL [µm]	Beta (SE)	*p*-value	Beta (SE)	*p*-value	Beta (SE)	*p*-value	Beta (SE)	*p*-value	Beta (SE)	*p*-value
HCVA [logMAR]	− 0.039 (0.007)	**< 0.001**	− 0.007 (0.003)	**0.02**	–	–	0.033 (0.008)	**< 0.001**	–	
LCVA [logMAR]	− 0.044 (0.008)	**< 0.001**	− 0.046 (0.009)	**< 0.001**	–	–	− 0.002 (0.012)	0.89	–	–
MD [dB]	0.762 (0.097)	**< 0.001**	0.449 (0.063)	**< 0.001**	–	–	− 0.317 (0.129)	**0.017**	–	–

	AQP4-IgG+		MOG-IgG+		MS		Interaction AQP4-IgG+ versus MOG-IgG+		Interaction AQP4-IgG+ versus MS	
GCIP [µm]	Beta (SE)	*p*-value	Beta (SE)	*p*-value	Beta (SE)	*p*-value	Beta (SE)	*p*-value	Beta (SE)	*p*-value
HCVA [logMAR]	− 0.037 (0.009)	**< 0.001**	− 0.011 (0.004)	**0.003**	− 0.042 (0.007)	**< 0.001**	0.025 (0.012)	**0.04**	− 0.010 (0.012)	0.44
LCVA [logMAR]	− 0.065 (0.010)	**< 0.001**	− 0.068 (0.012)	**< 0.001**	− 0.041 (0.010)	**< 0.001**	0.004 (0.016)	0.81	0.023 (0.014)	0.10
MD [dB]	0.835 (0.151)	**< 0.001**	0.571 (0.111)	**< 0.001**	0.515 (0.106)	**< 0.001**	− 0.270 (0.224)	0.23	− 0.302 (0.200)	0.13

Association between different measures of visual function and pRNFL- and GCIP-thickness respectively in a mixed linear spline model of NMOSD, MOGAD and MS eyes as well as interaction effects between the patient groups; all values are only from the sloped part of the spline for pRNFL and GCIP thickness values lower than 60 µm respectively (Fig. 2). No associations between pRNFL and visual function are provided for MS-eyes as there were not enough eyes below the 60 µm cutoff.

*AQP4-IgG*+ aquaporin-4 antibody positive NMOSD, *MOG-IgG*+ myelin-oligodendrocyte-glycoprotein associated disorder, *MS* multiple sclerosis, *SE* standard error, *HCVA* high contrast visual acuity, *LCVA* low contrast visual acuity, *MD* mean deviation, *pRNFL* peripapillary retinal nerve fiber layer, *GCIP* combined ganglion cell and inner plexiform layer.

Significant values are in bold.

Accordingly, below 60 µm of GCIP-thickness MOGAD eyes showed a less steep loss of HCVA than NMOSD eyes, with a similar trend for visual fields and no difference regarding loss of LCVA. Interestingly, there was no such difference between NMOSD and MS eyes. However, when inspecting the corresponding plot, one must note that the results for MS-eyes are heavily skewed by a single MS-ON eye with far outlying visual function. This eye belongs to a patient with a single severe ON episode leading to blindness. When excluding this eye, MS-eyes would show a trend of a less steep loss of HCVA than NMOSD-eyes (Beta = 0.022 SE = 0.012; *p* = 0.07).

**Figure 3:**
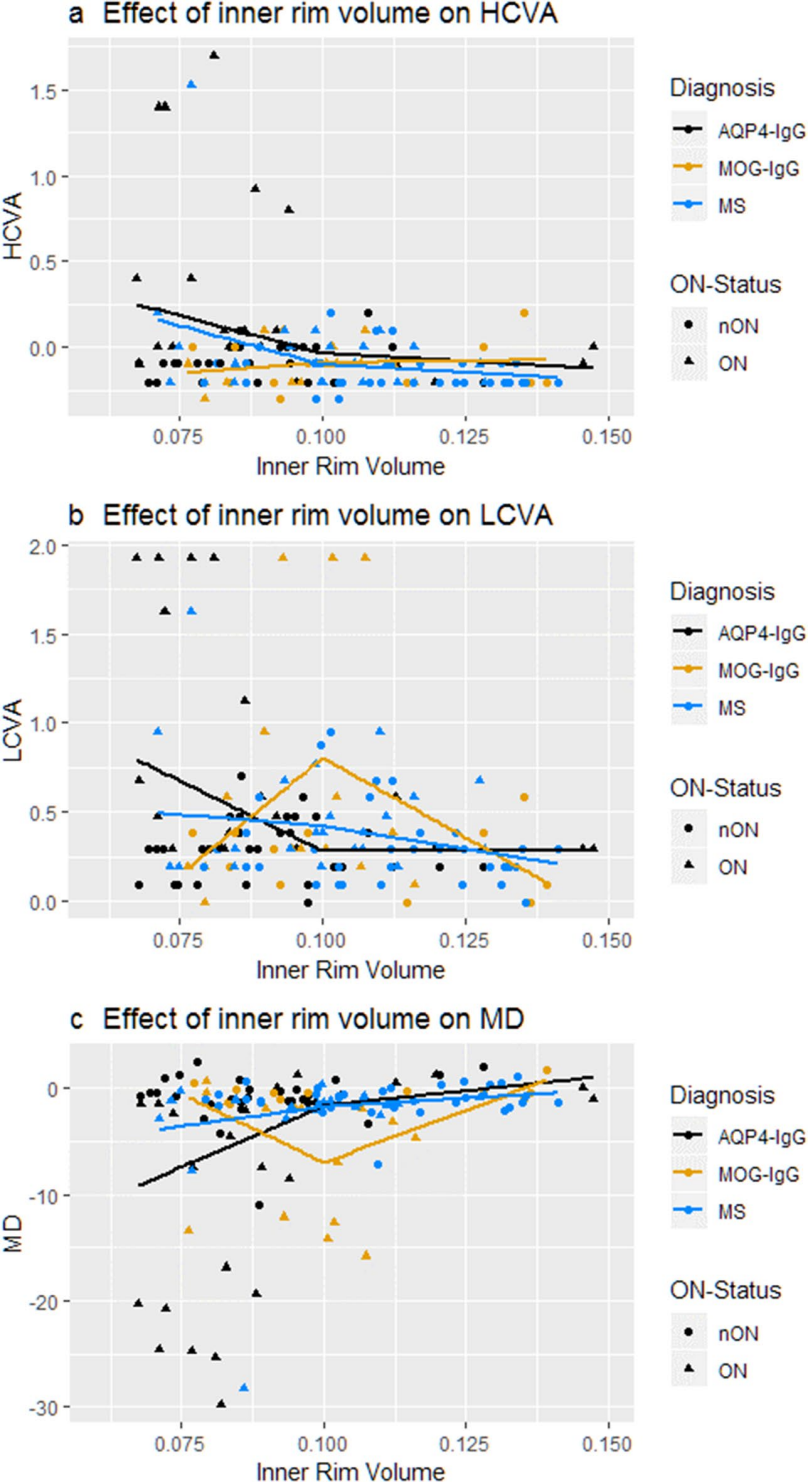
Association between visual function and foveal inner rim volume. Effect of inner rim volume on (**a**) high contrast visual acuity, (**b**) low contrast visual as well as (**c**) mean deviation of visual fields of patients` eyes. Cutoff for mixed linear spline model was visually identified at 0.1 mm^3^. AQP4-IgG: Aquaporin-4-IgG seropositive patients; MOG-IgG: Myelin-Oligodendrocyte-Glycoprotein-IgG seropositive patients; MS: Multiple Sclerosis patients; HCVA: High contrast visual acuity; LCVA: Low contrast visual acuity; MD: mean deviation.

None of the patient groups showed a relevant association between inner rim volume and any measure of visual function above the cutoff point of 0.1 mm^3^ (Fig. 3). However, the mixed linear spline model showed a significant association of inner rim volume and LCVA (Beta = − 20.55 ± 7.72; *p* = 0.01), MD of visual fields (Beta = 275.26 ± 123.10; *p* = 0.03) and—in trend—HCVA (Beta = − 11.14 ± 6.24; *p* = 0.08) in AQP4-IgG seropositive NMOSD eyes. MOGAD eyes did not show an identifiable pattern in the relationship between inner rim volume and visual function. MS eyes had a significant association between high contrast acuity and inner rim volume below the 0.1 mm^3^ cutoff (Beta = − 11.29 ± 4.86; *p* = 0.03), which was again entirely dependent on the outlier described above. When excluding this outlier, no significant association between inner rim volume of MS-eyes and any measure of visual function was found. ﻿In fe﻿male participants, only in the AQP4-IgG seropositive group significant associations were found (Supplementary Table 2).

### Association between inner rim volume and retinal neuro-axonal damage

**Figure 4:**
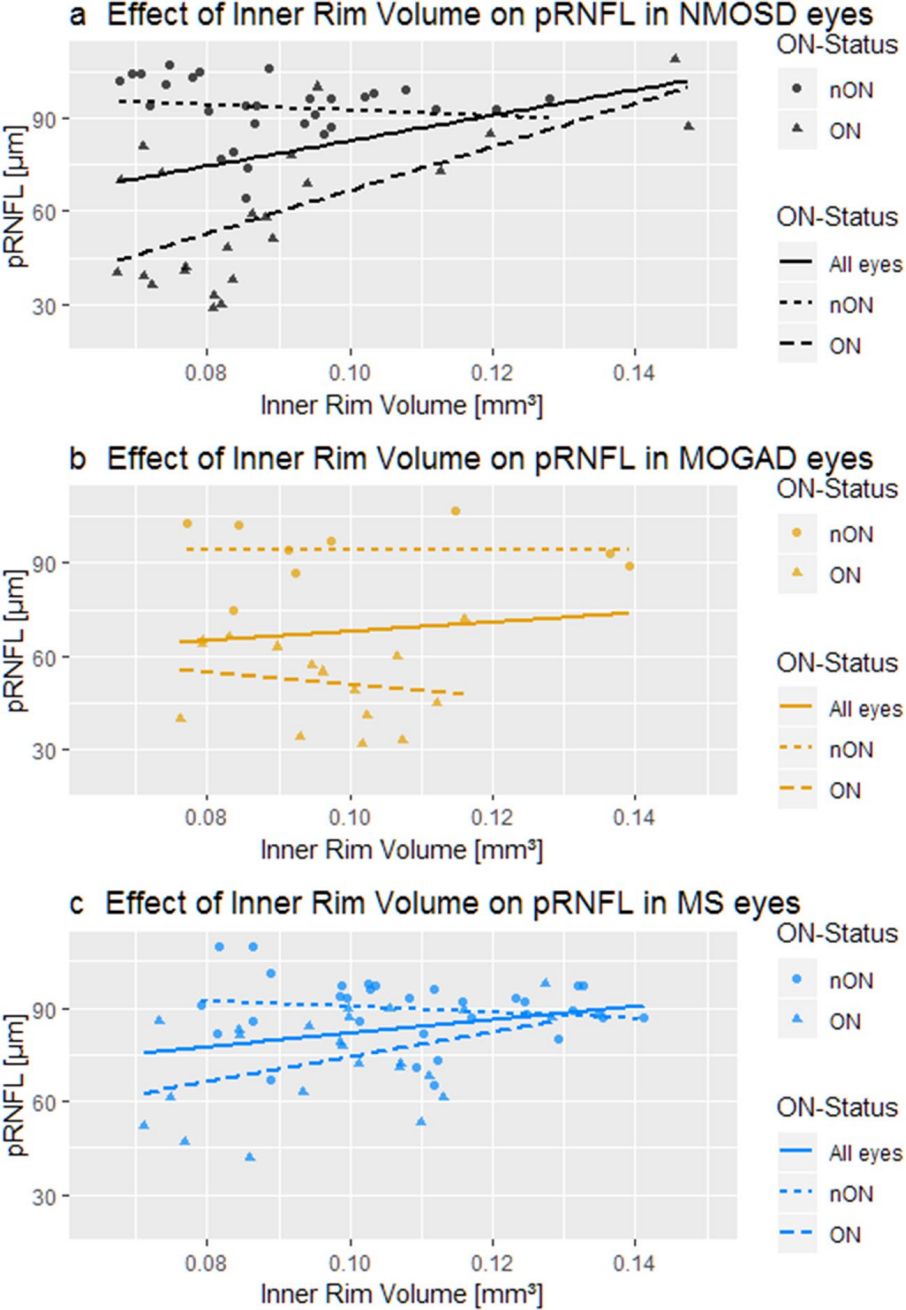
Association between inner rim volume and pRNFL in (**a**) NMOSD (Beta = 724.92 ± 208.93; *p* = 0.001), (**b**) MOGAD (Beta = 653.78 ± 329.25; *p* = 0.06) and (**c**) MS eyes (Beta = 315.02 ± 130.76; *p* = 0.02) AQP4-IgG: Aquaporin-4-IgG seropositive patients; MOG-IgG: Myelin-Oligodendrocyte-Glycoprotein-IgG seropositive patients; MS: Multiple Sclerosis patients.

To further identify the mechanism behind the greater loss of HCVA in NMOSD-eyes compared with MOGAD, we analyzed the relationship between pRNFL and foveal inner rim volume (Fig. 4). There was a positive association between inner rim volume and pRNFL in AQP4-IgG seropositive NMOSD eyes (Beta = 724.92 ± 208.93; *p* = 0.001, Fig. 4a), suggesting a simultaneous degradation of both over the course of the disease. MOGAD eyes showed a trend to a similar association (Beta = 653.78 ± 329.25; *p* = 0.06, Fig. 4b) while MS-ON eyes also showed a significant albeit smaller positive association (Beta = 315.02 ± 130.76; *p* = 0.02, Fig. 4c). However, if only ON-eyes were considered, there was a significant positive association between inner rim volume and pRNFL in NMOSD (Beta = 724.24 ± 206.56; *p* = 0.004) and to a lesser degree in MS (Beta = 507.79 ± 167.89; *p* = 0.007), but not in MOGAD (Beta = 241.82 ± 280.30; *p* = 0.41). In a subgroup analysis of only female participants, there was only a minor difference in effect size concerning the association between IRV and pRNFL (not shown).

In addition, inner rim volume was not associated with the number of ON in NMOSD (Beta = − 0.0009 ± 0.0009; *p* = 0.36) and MOGAD (Beta = − 0.0002 ± 0.0014; *p* = 0.91) but showed a very subtle association in MS-eyes (Beta = − 0.004 ± 0.002; *p* = 0.04). Disease duration had no significant effect on inner rim volume in either of the disease groups (NMOSD: Beta = − 2.66e^ − 6^ ± 1.21e ^− 5^; *p* = 0.83, MOGAD: Beta = − 2.66e ^− 6^ ± 3.21e^ − 5^; *p* = 0.42, MS: Beta = − 7.47e ^− 6^ ± 8.67e^ − 6^; *p* = 0.40).

## Discussion

In this study we analyzed the association between retinal structural damage including foveal changes and visual function in AQP4-IgG seropositive NMOSD patients and compared findings against MOGAD patients, MS patients and healthy controls. We found that (a) patients with AQP4-IgG seropositive NMOSD present with more impaired HCVA than MOGAD and MS despite similar retinal neuroaxonal damage; (b) in contrast, LCVA and MD are similarly affected in NMOSD, MOGAD and MS; (c) in all diseases the structure–function association followed a broken-stick model: there was no association between visual function and OCT above a certain threshold, but once a certain threshold was reached, more profound structural damage was associated with worse visual function. Here, HCVA and MD showed a steeper association with structural damage in NMOSD compared with MOGAD, with no significant difference when it comes to the rate of loss of LCVA; (d) Eyes from patients with NMOSD show additionally an association between foveal inner rim volume and visual function.

Several studies have investigated the relationship between visual and structural parameters in neuroinflammatory diseases. In MS and MOGAD, loss of visual field sensitivity does not follow a linear association with pRNFL thinning, but shows a drastic deterioration after a large amount of pRNFL is lost^28,29,31^. This is similar to how visual function relates to neuroaxonal damage in glaucoma, where this type of association has been termed *broken stick model*^35^. Alternatively, a linear association between visual acuity and retinal layer thickness has been proposed in MS^5,36^ as well as AQP4-IgG seropositive NMOSD and MOGAD^5^. In our study, the association of MD, HCVA and LCVA to pRNFL and GCIP thickness followed a broken stick model and not a linear association, with a threshold of approximately 60 µm for both pRNFL and GCIP. There was no such association for either disease group above 60 µm, supporting that neuroaxonal damage in the context of neuroinflammatory disease leads to loss of visual function, but only has a measurable effect once a certain amount of neuroaxonal content is lost. In other studies this cutoff point has been identified as being 50 µm for MOGAD^31^, 75 µm for MS^32,36^ or 60 µm for NMOSD^33^. Considering differences in methods and raters as well as the sizable interindividual differences of retinal layer thickness, these results are comparable.

Although the loss of retinal layers started to affect all patient groups at approximately the same threshold, NMOSD eyes suffered a steeper drop in HCVA and MD than MOGAD eyes. The less pronounced difference in the drop of visual fields is in line with a recent study that showed good HCVA outcome after MOGAD-associated ON, but worse visual fields outcomes^31^. The finding that each µm of retinal layer lost is worth more visual function in NMOSD than MOGAD is in line with a previous study, in which only eyes of NMOSD but not MOGAD patients showed an association between worse HCVA with lower GCIP^5^. Another study showed worse HCVA and stronger pRNFL thinning in MOGAD eyes at ON nadir, but also a higher recovery rate of visual function that resulted in comparable long term visual outcome in MOGAD and MS^37^. Together, our study and these others suggest that neuroaxonal damage alone cannot explain the differences in visual outcome between NMOSD patients and MOGAD or MS patients.

A model that might explain this discrepancy is AQP4-IgG driven Müller-cell dysfunction. AQP4-IgG seropositive NMOSD leads to a primary inflammatory astrocytopathy while demyelination is only a secondary process. MOGAD and MS on the other hand primarily result in inflammatory demyelination^38^. In the healthy retina, Müller cells are responsible for many trophic and regulatory functions, such as glucose metabolism, regulating blood flow as well as ion and water homeostasis^39^. Müller cells are found ubiquitously in the human retina, but are most dense in and around the fovea^10^. In NMOSD, Müller cells are a target of AQP4-IgG, which was demonstrated both in animal studies^40^ as well as NMOSD patients^41^. In vitro, AQP4-IgG causes non-inflammatory AQP4-receptor internalization and reduced proliferation in Müller cells^42^; in fact, Müller cell dysfunction has been directly demonstrated through specific ERG-waveforms in eyes of NMOSD patients^10^.

We found a significant association between the fovea’s inner rim volume and pRNFL in AQP4-IgG positive NMOSD eyes. Indeed, inner rim volume had a similar effect on visual function in NMOSD as pRNFL and GCIP thinning: After a certain amount of inner rim volume is lost (below 0.1 mm^3^), drastic deterioration of visual function begins. Regardless of underlying disease, inner rim volume in eyes with values > 0.1 mm^3^ were not associated with visual function. In NMOSD-ON eyes, there was also a strong positive association between foveal inner rim volume and pRNFL, which could not be found in MOGAD-ON eyes, suggesting AQP4-IgG transmitted Müller cell damage concomitant to neuroaxonal damage. Our results further suggest that the fovea’s contribution of vision is largest in HCVA, less noticeable in visual fields and seemingly absent in LCVA. This fits nicely to the test principles of each vision test, where HCVA is typically dominated by foveal vision, whereas in low contrast vision testing peripheral rods dominate, and visual field testing comprises an average over central and peripheral retinal areas.

In our study, MS patients’ eyes behaved in a similar fashion as NMOSD eyes in regard to neuroaxonal damage but surprisingly also foveal inner rim volume, yet to a lesser degree. While the association of visual function to pRNFL and GCIP in the MS group was dependent on a single outlying eye, there was also a similar association between inner rim volume and pRNFL, marking a clear difference to the MOGAD and control group. A primary retinopathy has been reported in MS by one study, which could explain this association. Alternatively, the density of the retina’s superficial and deep capillary plexus is reduced in MS patients, making an indirect effect through foveal microcirculation possible^43^.

An important strength of our study is the availability of a comprehensive suite of visual tests, which reports HCVA, LCVA and MD. Our study has several important limitations, most notably the small sample size. This is problematic for foveal parameters, which show large interindividual variation even in healthy eyes^44^.

Another problem is the significant difference in age between the NMOSD and MS group, which we decided to not correct, because the effect of age on retinal layers is subtle^19^ and given the low sample size we did not want to overstrain the models. Further, vision assessment was carried out in one single session, but at least two independent measurements of visual performance are recommended to account for daily variation in performance of patients. Another concern may be that, the minimum timeframe after ON of 3 months that we set as an inclusion criterion might be too short, especially when considering MOGAD, in which further changes to pRNFL have been shown to occur within 6 months from clinical attacks other than ipsilateral ON^45^. On the other hand, another study did not show further thinning of pRNFL and GCIP 6 months after ON in MS and NMOSD when compared to the respective thickness after 3 months^46^. A further potential confounder may be different numbers of ON in the different cohorts. While the number of ON was not significantly different between MOGAD and NMOSD patients, we cannot fully rule out that non-linear effects of the number of ON, which have been reported both for NMOSD and MOGAD^47,48^, may have influenced the result. Because most patients in our study had multiple ON episodes, we were not able to perform an analysis in eyes with only one ON.

Although our results indicating a potential contribution of Müller cells to high contrast vision loss are promising, the findings need to be interpreted with caution considering the low sample size and the other above-mentioned limitations. Important other contributors should be alternatively considered and investigated, most notably optic nerve demyelination and retinal vascular changes. Visual evoked potentials (VEP) could elucidate how optic nerve demyelination affects visual outcome, and VEP latency changes were indeed reported recently in NMOSD even in absence of ON^49^. Alternatively, the size of the foveal avascular zone (FAZ) as measured by OCT angiography is already of interest in several other ophthalmological diseases^50,51^. The possible importance of foveal microcirculation in the development of visual impairment in NMOSD becomes especially apparent when considering the trophic role of Müller cells and the high expression of AQP4 in foot processes facing blood vessels^52^.

## Supplementary Information

Supplementary Information.

## Data Availability

As patient consent did not cover publication of individual data, the data used in this manuscript will be shared on reasonable request from corresponding author.
