# Supplementary material for: Structure–function correlates of vision loss in neuromyelitis optica spectrum disorders
Source: Sci Rep. 2022 Oct 20;12:17545. doi: 10.1038/s41598-022-19848-4 (PMC9585067; doi:10.1038/s41598-022-19848-4)
Supplement: Supplementary file 1 — Supplementary Information. [file 41598_2022_19848_MOESM1_ESM.docx]

Supplemental Table 1

|  | AQP4-IgG+ | | MOG-IgG+ | | MS | |
| --- | --- | --- | --- | --- | --- | --- |
| pRNFL  [µm] | **Beta (SE)** | **p-value** | **Beta (SE)** | **p-value** | **Beta (SE)** | **p-value** |
| HCVA  [logMAR] | -0.001 (0.003) | 0.75 | 0.001 (0.003) | 0.31 | -0.002 (0.002) | 0.33 |
| LCVA [logMAR] | -0.006 (0.004) | 0.09 | 0.003 (0.005) | 0.53 | -0.004 (0.002) | 0.05 |
| MD [dB] | 0.061 (0.049) | 0.21 | 0.038 (0.031) | 0.23 | 0.006 (0.024) | 0.8 |
| GCIP[µm] | **Beta (SE)** | **p-value** | **Beta (SE)** | **p-value** | **Beta (SE)** | **p-value** |
| HCVA [logMAR] | -0.002 (0.012) | 0.86 | 0.008 (0.005) | 0.15 | 0.003 (0.005) | 0.60 |
| LCVA [logMAR] | 0.002 (0.013) | 0.86 | 0.023 (0.017) | 0.20 | -0.004 (0.006) | 0.56 |
| MD [dB] | 0.069 (0.211) | 0.75 | 0.059 (0.157) | 0.71 | -0.042 (0.081) | 0.61 |

Association between different measures of visual function and pRNFL- and GCIP-thickness respectively in a mixed linear spline model of NMOSD, MOGAD and MS eyes all values are only from the sloped part of the spline for pRNFL and GCIP thickness values higher than 60µm respectively (Figure 2). AQP4-IgG+: Aquaporin-4 antibody positive NMOSD; MOG-IgG+: Myelin-Oligodendrocyte-Glycoprotein associated disorder; MS: Multiple Sclerosis; SE: Standard error; HCVA: High contrast visual acuity; LCVA: Low contrast visual acuity; MD: mean deviation; pRNFL: peripapillary retinal nerve fiber layer; GCIP: combined ganglion cell and inner plexiform layer

Supplemental Table 2

|  | AQP4-IgG+ | | MOG-IgG+ | |
| --- | --- | --- | --- | --- |
| Inner rim volume  [mm³] | **Beta (SE)** | **p-value** | **Beta (SE)** | **p-value** |
| HCVA  [logMAR] | -12.27 (6.11) | 0.051 | -1.95 (5.66) | 0.74 |
| LCVA [logMAR] | -20.70 (7.82) | **0.01** | 13.62 (19.69) | 0.51 |
| MD [dB] | 242.03 (116.47) | **0.04** | -222.41 (129.41) | 0.13 |

Association between different measures of visual function and inner rim volume in a mixed linear spline model of only female eyes of the NMOSD- and MOGAD-group. All values are only from the sloped part of the spline for inner rim volume values lower than 0.1mm³. AQP4-IgG+: Aquaporin-4 antibody positive NMOSD; MOG-IgG+: Myelin-Oligodendrocyte-Glycoprotein associated disorder; MS: Multiple Sclerosis; SE: Standard error; HCVA: High contrast visual acuity; LCVA: Low contrast visual acuity; MD: mean deviation

Supplemental Figure 1


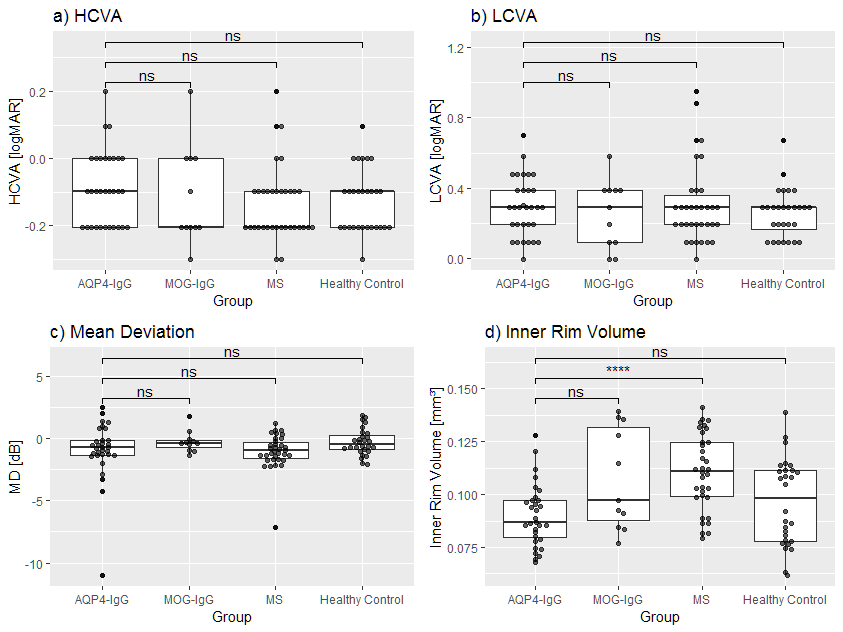


1. High contrast visual acuity b) Low contrast visual acuity c) mean deviation of visual fields and d) inner rim volume of the fovea of eyes without history of ON AQP4-IgG: Aquaporin-4 IgG seropositive patients; MOG-IgG: Myelin-Oligodendrocyte-Glycoprotein seropositive patients; MS: Multiple Sclerosis; HCVA: High contrast visual acuity; LCVA: Low contrast visual acuity; logMAR: Logarithm of minimum angle of resolution; MD: mean deviation; p-values from Mann-Whitney-U Test (ns: p > 0.05; *: p < 0.05; **: p < 0.01; ***: p < 0.001; ****: p < 0.0001)
